# Fine mapping of V(D)J recombinase mediated rearrangements in human lymphoid malignancies

**DOI:** 10.1186/1471-2164-14-565

**Published:** 2013-08-19

**Authors:** Eitan Halper-Stromberg, Jared Steranka, Nicolas Giraldo-Castillo, Timothy Fuller, Stephen Desiderio, Kathleen H Burns

**Affiliations:** 1 McKusick-Nathans Institute of Genetic Medicine, Johns Hopkins University, Baltimore, MD, USA; 2 Department of Molecular Biology and Genetics, Johns Hopkins University School of Medicine, Baltimore, MD, USA; 3 Department of Oncology and The Sidney Kimmel Comprehensive Cancer Center, Johns Hopkins Hospital, Baltimore, MD, USA; 4 Immunology Unit of the Institute for Cell Engineering, Johns Hopkins University School of Medicine, 720 Rutland Avenue, Ross Building, Room 524, Baltimore 21205, MD, USA; 5 Department of Pathology, Johns Hopkins University, Baltimore, MD, USA; 6 Department of Pathology, Johns Hopkins School of Medicine, 720 Rutland Avenue, Ross Building, Room 524, Baltimore 21205, MD, USA; 7 Los Andes University, Bogotá, Colombia; 8 Mercer University School of Medicine, Macon, GA, USA

**Keywords:** V(D)J recombination, Oncogenic translocation, Lymphoid tumors, MYC, BCL-2, V replacement

## Abstract

**Background:**

Lymphocytes achieve diversity in antigen recognition in part by rearranging genomic DNA at loci encoding antibodies and cell surface receptors. The process, termed V(D)J recombination, juxtaposes modular coding sequences for antigen binding. Erroneous recombination events causing chromosomal translocations are recognized causes of lymphoid malignancies. Here we show a hybridization based method for sequence enrichment can be used to efficiently and selectively capture genomic DNA adjacent to V(D)J recombination breakpoints for massively parallel sequencing. The approach obviates the need for PCR amplification of recombined sequences.

**Results:**

Using tailored informatics analyses to resolve alignment and assembly issues in these repetitive regions, we were able to detect numerous recombination events across a panel of cancer cell lines and primary lymphoid tumors, and an EBV transformed lymphoblast line. With reassembly, breakpoints could be defined to single base pair resolution. The observed events consist of canonical V(D)J or V-J rearrangements, non-canonical rearrangements, and putatively oncogenic reciprocal chromosome translocations. We validated non-canonical and chromosome translocation junctions by PCR and Sanger sequencing. The translocations involved the *MYC* and *BCL-2* loci, and activation of these was consistent with histopathologic features of the respective B-cell tumors. We also show an impressive prevalence of novel erroneous V-V recombination events at sites not incorporated with other downstream coding segments.

**Conclusions:**

Our results demonstrate the ability of next generation sequencing to describe human V(D)J recombinase activity and provide a scalable means to chronicle off-target, unexpressed, and non-amplifiable recombinations occurring in the development of lymphoid cancers.

## Background

In humans, the number of unique immunoglobulin (Ig) and T-cell receptors (TCRs) approaches 10^12^[1]. Much of this diversity is generated through the combinatorial assembly of antigen receptor genes from discrete DNA segments by the process of V(D)J recombination. Aberrant V(D)J recombination is one mechanism responsible for chromosomal translocations associated with lymphoid malignancies. Characterization of canonical and aberrant V(D)J rearrangements is fundamental to understanding the primary antigen receptor repertoire and the pathogenesis of lymphoid malignancies. Reflecting this interest and developments in sequencing technology, the International Immunogenetics Information System (IMGT) blast database has increased in size from ~21 Mb to ~63 Mb of human sequence data since 2003 [2]. Even at this size, however, the database comprises only 746 functionally recombined human alleles and 321 non-functional alleles or pseudogenes.

Identification of V(D)J recombination products by next generation sequencing techniques is an attractive way to obtain a more comprehensive sample of the estimated repertoire [3]. However, sequence properties that define Ig and TCR loci also make their interpretation from short read technology challenging. Standard practices like removal of poorly aligned reads must be modified to accommodate the specific structural changes expected during V(D)J recombination and the prevalence of regional segmental duplications. Complexities associated with this task are well recognized [4].

We have developed a targeted method to selectively sequence V(D)J recombination events. It has the advantage of revealing off-target and non-canonical rearrangements that may be unexpressed or unamplifiable with primer pairs designed to detect canonical rearrangements. We demonstrate the method in a panel of primary lymphoid tumors and lymphoid cell lines and report high-resolution sequences comprising canonical and unknown, non-canonical rearrangements as well as two chromosome translocations mediated by erroneous V(D)J recombination events. We expect that this approach will provide deeper insight into the role V(D)J recombination plays in pathogenesis of lymphoproliferative disease.

## Results

### Experimental approach

**Figure 1:**
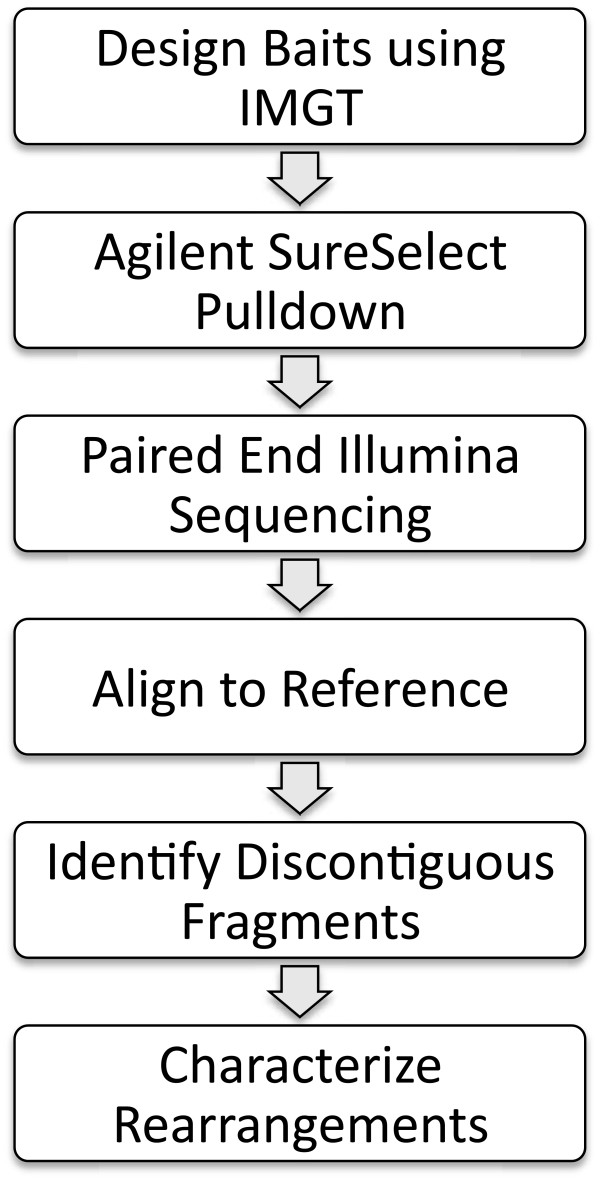
Outline of assay and analysis pipeline.

Genomic DNA fragments were enriched for recombination signal sequences (RSS) using an RNA bait hybridization based approach and subjected to paired-end Illumina sequencing (Figure 1). Total reads per sample ranged from 434 k to 797 k. Reads were aligned to the reference genome assembly (hg19), and read pileups were seen within 1 kb intervals surrounding bait sequences. The most distant rearrangement observed and validated contained reads extending about 255 bp away from the edge of the nearest bait. Between 83% and 94% of total reads aligned to the reference genome, and approximately 50% of these mapped to targeted regions (range 38%-54%).

### Repetitive sequences

Ambiguous alignments (i.e., reads matching multiple genomic positions) were frequently encountered. This reflects inherent properties of antigen receptor loci, which contain arrays of homologous gene segments at several genomic loci. Baits and surrounding contexts were enriched for segmental duplications about 6-fold above the genome-wide level. Where possible we resolved ambiguous alignments with sensitive realignment. We frequently could validate events within segmental duplications, provided the two junctional sequences were not homologous. Of the 8 non-canonical events reported here, 4 were within segmental duplications, and of the 18 canonical events, 9 were within segmental duplications. A more detailed description of how we distinguished true structural variants from alignment artifacts over these intervals and an associated R package to aid in visualizing alternate alignments will be described in a companion paper (Halper-Stromberg, et al. In preparation).

### Canonical V(D)J and V-J events

**Figure 2:**
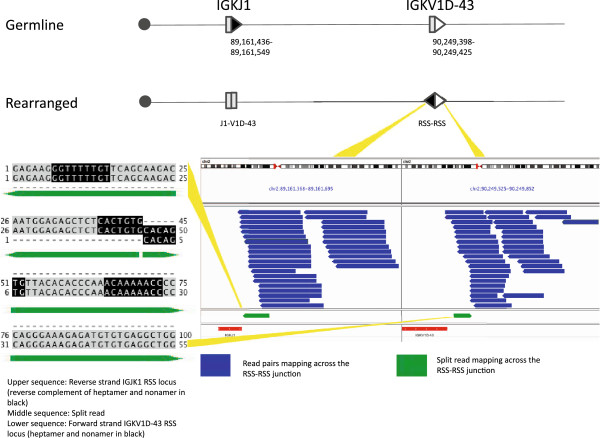
**A canonical V-J rearrangement of the kappa immunoglobulin light chain locus of the LCL.** The rearrangement occurred by inversion of a >1 Mb segment of 2p11.2. A schematic of the initial allele and the resulting product of the recombination are shown at the top. V and J segments are shown as gray boxes; recombination signal sequences (RSSs) are shown with arrows and marked with their corresponding genome coordinates. Arrow orientation reflects the direction of the RSS (heptamer-to-nonamer), filled arrows illustrate RSS with 23 bp (two turn) spacers, and open arrows illustrate 12 bp (one turn) spacers. The centromere side is marked with a circle, not distanced to scale. The RSS-RSS junction was detected by the sequencing strategy (right lower panel); blue reads denote pairs mapping across the junction. V and J segments are shown in red below the reads. The rearrangement was confirmed by Sanger sequencing, with the recovered sequence matching the junction spanning sequence found with a split-reads (left lower panel, middle sequence). The split-reads sequence is shown aligned to the two sequences present in the reference genome (hg19). The RSS heptamers and nonamers are shown in black. The IGKJ1 RSS is shown reverse complemented while the IGKV1D-43 is shown in proper 5*'* to 3*'* orientation, reflecting the nature of a split-reads over an inversion.

Normal V(D)J and V-J recombination juxtaposes coding sequences for immunoglobulin or T-cell receptor proteins by deletion or inversion of intervening gDNA. We observed 18 canonical events in this study. We defined 14 of these at single base resolution using split-reads, 10 precisely defined rearrangements directly over coding sequence and 4 precisely defined at the RSS-RSS junctions created via inversion (Figure 2). The remaining 4 we inferred from paired read pileups only. Of the 10 immunoglobulin events defined to the base, 2 were IGK inversions and 8 were IGH deletions. Both IGK inversions and 4 IGH deletions were in-frame, productive rearrangements based upon the ORF annotation of V(D)J elements from NCBI and Ensembl. The other 4 IGH deletions were unproductive rearrangements, 3 out of frame and 1 in frame but containing only 35 bp of a 175 bp IGK coding segment.

Of the 18 canonical recombinations observed, half reflected immunoglobulin heavy chain (IGH) locus deletions. Most of these were observed in a single EBV-transformed lymphoblastoid B-cell line (LCL, HuRef DNA), whereas no more than two rearranged IGH alleles were seen in monoclonal B-cell proliferations (Table 1). This is consistent with the reported oligoclonality of lymphoblastoid cells [5]. Although we did not see recurrent use of any of the more numerous IGH V or IGH D segments, we did note repeated usage of J6 and J4 segments. This is not surprising given the relative numbers of each segment type. Out of 6 IGH D-J deletions in LCL, J6 was used 4 times and J4 was used once. Out of 3 IGH D-J deletions in the B-cell neoplasms, J6 was used once and J4 was used twice. Our observations are congruent with previous reports indicating J4 and J6 over representation in multiple B cell contexts including peripheral B lymphocytes and B-ALL [6].

**Table 1:** Observed V(D)J recombination events

	**V-D-J**	**V-J**	**D-J**	**V-D**	**V-V**	**t(X;X)**
Sample						
A) pre-B ALL		(1) IGH*				
		(1) IGK				
MCL		(2) IGL	(1) IGH			
RAMOS		(1) IGK	(1) IGH			
B) chronic T-Cell leukemia		*(1) TRB*				
DB					*(1) IGH*	*t(14;18)*
ARH-77					*(1) IGH*	
C) LCL		(2) IGK	(5) IGH	(1) IGH	*(1) IGH*	
					*(1) IGK*	
Loucy	(2) TRB			*(1) IGH*		
Burkitt-like lymphoma		(1) IGK				*t(2;8)*

(A) Samples for which only canonical V-D-J recombination was observed. ^*^The V-J rearrangement observed in the pre-B ALL is likely to be normal V-D-J, however we obtained full read alignments only for read pairs mapping one end to IGHJ4 and the other to IGHV4-34. (B) Samples for which only non-canonical events were observed. Non-canonical events are indicated as italicized text. (C) Samples for which both canonical and non-canonical events were observed.

The immunoglobulin kappa light chain locus (IGK) and the locus encoding T-cell receptor β chain (TRB) loci followed the same pattern, with recurrent utilization of J segments but not of the more numerous V segments (IGK) or V or D segments (TRB). The IGK locus J1 and J2 RSSs were recombined one time each in the LCL, out of 2 observed V-J joints, and once and 2 times in neoplastic B-cells, respectively, out of 3 observed V-J joints. Use of TRB J2 was observed in two of three T-cell neoplasms studied. We observed two events using the same variable segment (V3-19) at the immunoglobulin lambda light chain locus (IGL); these were recombined with different J elements.

Most (6/9) canonical, non-IGH rearrangements detected were inversions. We attribute this to a bias of our method towards inversion detection. Capturing inversional rearrangements, which retain RSS sequences, is an expected outcome as probes were designed to hybridize RSS sequences directly. Deletional rearrangement detection, conversely, requires sequencing rearranged segments adjacent to a captured RSS not participating in the rearrangement.

Four of the inversion junctions observed represented signal joints and two represented coding joints. One coding joint resulted in a productive V-J allele [7]. The other coding joint resulted in a productive V(D)J allele [8]. Four of the 6 inversions occurred in a single sample, 3 in neoplastic B-cells, and 1 in a T cell neoplasm. The LCL sample exhibited 2 inversions, both at IGK, one of these being the largest intra-chromosomal rearrangement observed (Figure 2). This larger inversion involved a >1 Mb segment of 2p11.2 bringing IGKJ1 and IGKV1D-43 together.

All five canonical rearrangements in the IGK locus were inversions, including one in a Burkitt-like lymphoma using a J element < 1 Kb centromeric of the t(2;8) breakpoint seen on a different allele of the same sample (Additional file 1: Figure S1). This event involved a 740 Kb segment of 2p11.2, bringing IGKJ2 together with IGKV1D-39. The T-cell inversion in Loucy cells was the only inversional joining of V, D, and J segments that we observed (Additional file 1: Figure S2). This rearrangement we infer occurred in 2 steps: a deletion bringing TRBJ2-1 into contact with an upstream D element in 7q34, and the subsequent joining of the DJ unit to TRBV5-6 via inversion.

### Non-Canonical and lineage inappropriate rearrangements

**Figure 3:**
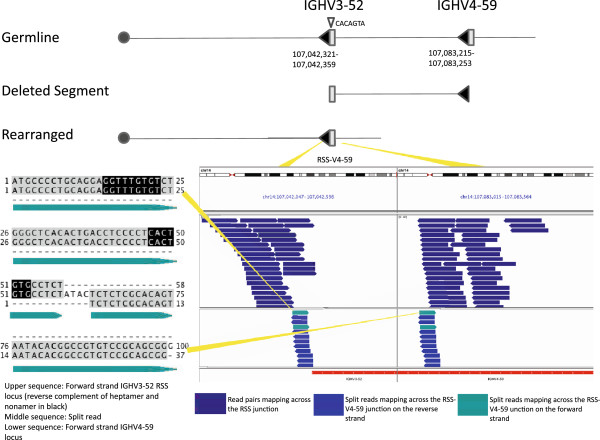
**An interstitial deletion within the IGH locus variable domain of the DB cell line.** The deletion involves a 41 kb segment flanked by two RSS sequences with 23 bp spacers (filled arrows). The deletion was likely mediated through V replacement, although this allele has not undergone prior rearrangement at this locus. The deletion is initiated through recognition of an oppositely oriented cryptic heptamer (shown as a small downward facing, open triangle) within IGHV3-52 by the RSS of IGHV4-59. The deletion was detected by sequencing of the RSS-V4-59 junction (lower right panel); blue reads denote sequences with read pairs mapping across the junction. The rearrangement was confirmed by Sanger sequencing, with the recovered sequence matching the junction spanning sequence found with a split-reads (left lower panel, middle sequence). The split-reads sequence is shown aligned to the two sequences present in the reference genome (hg19). Nucleotides aligning to the reference regions are shown in gray, N-region nucleotides are white, and the reverse complement of the retained heptamer and nonamer of IGHV3-52 are shown in black.

Four non-canonical events were interstitial deletions bringing two V elements together: the RSS of one V segment and the coding sequence of a second V segment. The RSS of the invading V appeared to use a cryptic heptamer sequence within the removed V and at the signal end. This has previously been described as V replacement when it involves recombination between a germline V segment and a previously assembled V_H_DJ_H_ or V_L_J_L_ unit [9]. Our method enabled observation of this phenomenon at both IGH and IGK loci and without prior rearrangement of the replaced V (Figure 3, Additional file 1: Figures S3, S4 and S5). Three V-to-V events occurred within IGH, all utilizing V4-59 with either V1-58 or V3-52; the remaining example occurred at IGK. Two of these events, one at IGH and one at IGK were seen in the LCL. We aligned split-reads and Sanger sequences for these regions to the build of HuRef DNA, and see no evidence that this was a germline or constitutional event. Of the three V4-59 deletions, all appear to have occurred independently in the lymphoid lineage with unique insertions of N-region nucleotides by terminal deoxynucleotidyl transferase (TdT). Two occurred in tumor cell lines, ARH-77 and DB; in each case small portions of the coding segment between the centromeric RSS and the cryptic heptamer were retained adjacent to N-region nucleotides at the junction.

Of the other two non-canonical events, one was a lineage-inappropriate rearrangement, a V to D rearrangement via deletion at IGH in Loucy cells, which are derived from a precursor T-cell acute lymphoblastic leukemia [10]. The other non-canonical recombination was a putative V-J rearrangement at TRB in a chronic T-cell leukemia sample, apparently lacking the expected D element between the V and J (Additional file 1: Figure S6). This event involved a 16 Kb inversion in 7q34 rearranging TRBJ2-1 with TRBV30. We obtained sequence from the V RSS to J RSS junction but not the V-J segment junction, inferring that neither of the two TRBD elements had recombined with J elements from the TRBJ2 cluster preceding the inversion, as this would have deleted the 12 bp spacer RSS flanking TRBJ2-1 that we observed. The recombination did not violate the 12–23 recombination rule, but a D-J rearrangement should have preceded incorporation of the V segment [11].

### N-Region nucleotides

We observed addition of N-region (non-coded) nucleotides at all seven validated junctions involving at least one coding segment. These included 4 short additions between 2 and 5 bp across 3 samples, 2 in LCL, 1 in DB cells, and 1 in Loucy cells; 2 intermediate size additions of 8 and 11 bp both in DB cells; and one long addition of 23 bp in ARH-77. The activity of terminal deoxynucleotidyl transferase (TdT) enzyme can explain these additions, although the addition in ARH-77 is unusually long. N regions were highly specific to coding segments and not seen at any of the 6 RSS-RSS junctions in which we obtained split-reads, including the five IGK canonical inversions and the V to J rearrangement at TRB in the chronic T-cell leukemia sample.

### Oncogenic interchromosomal translocations

In two samples, large numbers of reads mapped to loci on other chromosomes not targeted for sequencing, reflecting possible V(D)J recombinase mediated translocations with breakpoints near RSS. The first off-target sequences mapped on the telomeric side of the *MYC* oncogene on chromosome 8 and occurred with partial read or read pairs mapping on the centromeric side of IGKJ4 on chromosome 2. This was consistent with a t(2;8) translocation. This lesion was observed in a pediatric patient who had been immunosuppressed after receiving an organ transplant, and is consistent with having contributed to the development of a Burkitt-like lymphoma in this person. Genomic DNA from fresh frozen primary cells was used for our analysis.

**Figure 4:**
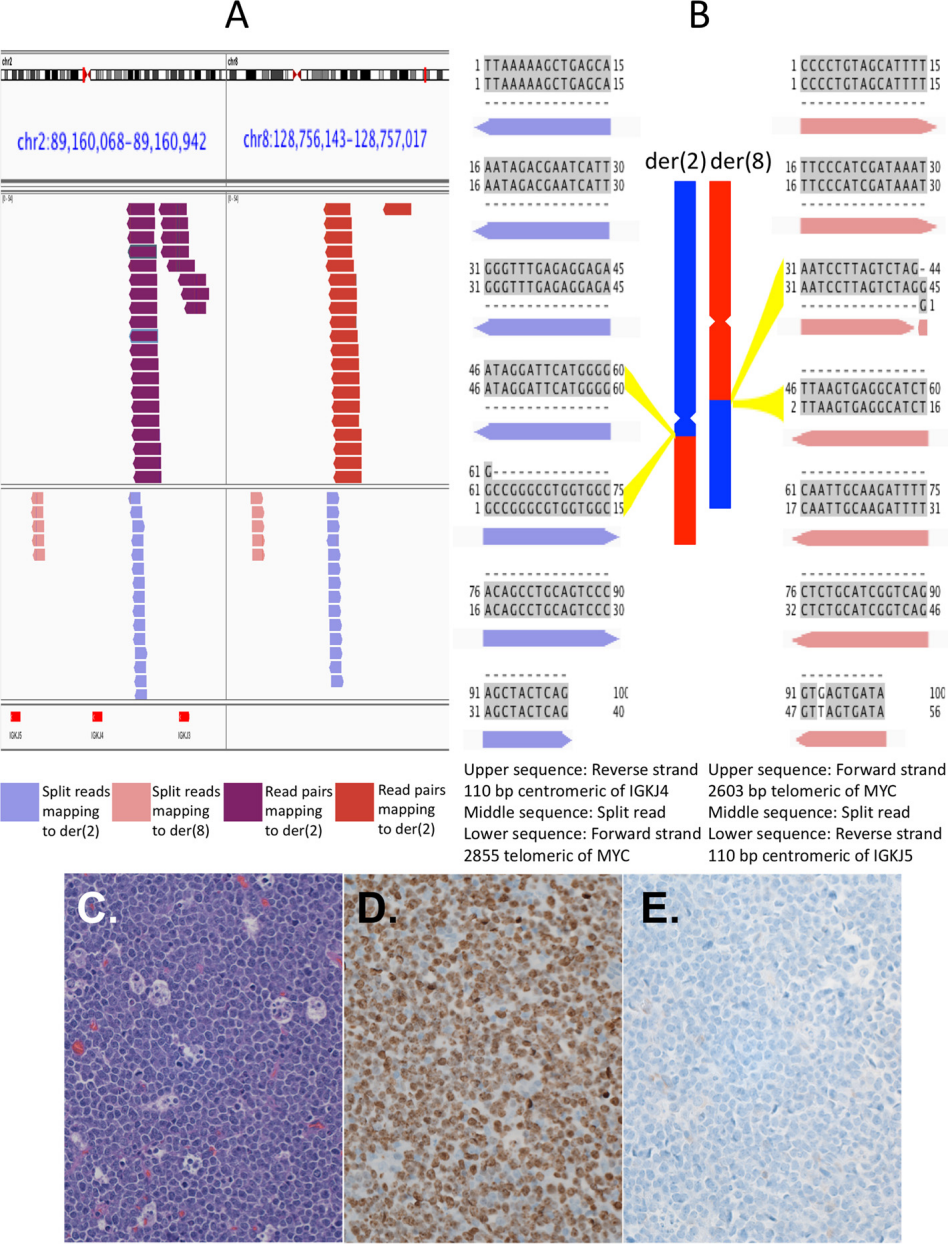
**Derivative chromosomes for the reciprocal t(2;8) IGK-*MYC *translocation in Burkitt-like lymphoma. (A)** Full length reads mapping to IGK and *MYC* loci on der(2) and split-reads mapping to IGK and *MYC* loci on both der(2) and der(8). Red segments in the bottom panel indicate IGKJ segments. Full length reads all map to the negative strand indicative of inversion. Split-reads map in opposite orientations also consistent with inversion. The gaps between pink and blue split-reads pileups in the left and right panels cover sequence deleted before recombination. This includes a 252 bp deletion from der(8) and a 317 bp deletion from der(2). **(B)** The rearrangements were confirmed by Sanger sequencing, with the recovered sequences matching the junction spanning sequences found with split-reads. The split-read sequences are shown aligned to the two sequences present in the reference genome (hg19) from each junction. Aligned nucleotides are shown in gray and a mismatch between the reference and the sample is shown in white. **(C)** This tumor had many histologic features of a Burkitt lymphoma, consistent with activation of the *MYC* oncogene. A hematoxylin and eosin stained section shows a monotonous population of round, medium sized B-cells with large nuclei and variable cytoplasmic retraction. Intermixed macrophages with cellular debris (tingible body macrophages) impart a starry sky appearance. Numerous mitotic figures were seen. **(D)** Immunohistochemistry showed nuclear Ki67 proliferation antigen staining in a high percentage of the cells (brown). **(E)**. The cells showed a mature germinal center cell phenotype with CD20, CD10, BCL6, restricted expression of kappa surface immunoglobulin, and no TdT. They were not immunoreactive for BCL2 (shown; blue counterstain).

Upon investigation with split-reads, we determined junctional sequences for both der(2) and der(8). The der(8) breakpoint occurred 110 bp centromeric of IGKJ5 and 2603 bp telomeric of *MYC*. The der(2) breakpoint was 110 bp centromeric of IGKJ4 and 2855 bp telomeric of *MYC* (Figure 4 A,B). These breakpoints indicated a 252 bp loss of sequence from der(8), and 317 bp loss from der(2). *MYC*-IGK translocations are not uncommon in Burkitt lymphoma, occurring with a 5-10% frequency. Numerous locations downstream of *MYC*, even hundreds of Kb away, have been reported as breakpoints in *MYC* activating *MYC*-IGK translocations. Pathologic features in our case are consistent with *MYC* activation (Figure 4C-E). *MYC*-IGK breakpoints near ours have been reported. In a recent report of breakpoints for 10 high-grade lymphoma samples with t(2;8), one patient sample and one cell line had der(8) breakpoints between IGKJ4 and IGKJ5, similar to our IGK der(8) breakpoint [12]. One patient sample in their cohort showed a 435 bp deletion from der(2), similar in size to our der(2) deletion. One cell line from their cohort exhibited loss of sequence from both der(2) and der(8), although shorter than what we observed (29 and 57 bp, respectively).

**Figure 5:**
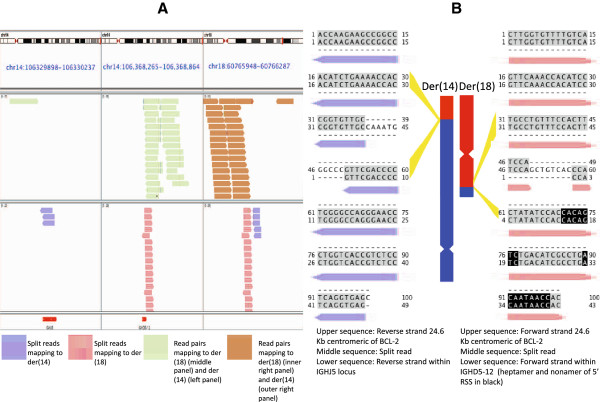
**Derivative chromosomes for the t(14;18) IGH-BCL2 translocation in the DB cell line. (A)** Full length and split-reads mapping to both der(14) and der(18). Der(14) reads map to the *BCL-2* and IGHJ5 loci and der(18) reads map to the *BCL-2* and IGHD5-12 loci. Red segments in the bottom panel indicate IGH D and J segments. The small gap between pink and blue split-read pileups in the right panel covers 12 bp of sequence deleted before recombination. This junction was complex with deletion of 12 bases and insertion of 8 (shown in B, right side). The panel on the left is 38.5 Kb centromeric of the middle panel, and is shown as a separate panel because the intervening sequence was deleted from der(14). **(B)** The rearrangements were confirmed by Sanger sequencing, with the recovered sequences matching the junction spanning sequences found with split-reads. The split-read sequences are shown aligned to the two sequences present in the reference genome (hg19) from each junction. Aligned nucleotides are in gray, N-regions are in white, and the heptamer and nonamer of IGHD5-12 are shown in black. Both junctions contain N-regions, 11 N-region nucleotides on der(14) and 8 N-region nucleotides on der(18).

The second interchromosomal translocation occurred in the DB cell line, derived from a diffuse large B-cell lymphoma (DLBCL). The t(14;18) is a common translocation for diffuse large B-cell lymphomas, occurring in about 20% of cases [13]. These resemble follicle center cells; expression of CD10, which had been reported for this cell line, is consistent with that origin. We observed an unbalanced translocation with loss of 38,492 bp from IGH on der(14), between IGHJ5 and IGHD5-12, and loss of 12 bp from der(18), 24.6 Kb centromeric of *BCL-2* (Figure 5A and B). Junctional sequences for both der(14) and der(18) contained N-regions, 8 on der(18) and 11 on der(14). Loss of sequence between the IGHJ and IGHD loci on der(14) has been previously reported in the DLBCL line, SU-DHL-6, as well as B cell lymphoma patient samples [14]. Short junctional additions on der(14) and der(18) are also reported for SU-DHL-6.

Previously described IGH-*BCL-2* translocation breakpoints in B-cell neoplasms are similar to ours but not exactly matching. We searched 2 databases to determine the novelty of our breakpoints; breaks within IGHJ5 are common and closely match ours, whereas the other breakpoints involved are less often seen and do not match as closely. In dbCRID: Database of Chromosomal Rearrangements in Disease, we found sequence at the breakpoints on der(14) for 6 diffuse large B-cell lymphoma patient [15]. On the centromeric side, these clustered in a 22 bp window within IGHJ6, approximately 600 bp centromeric to our breakpoint within IGHJ5 [16]. On the telomeric side, breakpoints clustered in a 50 bp window within the 3′ UTR of *BCL-2*, approximately 27.5 Kb telomeric of our breakpoint.

IGH-*BCL-2* translocation breakpoints closer to the ones we observed were found in a chromosomal rearrangement breakpoint database containing 551 t(14;18) entries [17]. We found 2 exact matches to our der(14) breakpoint on the centromeric side (within the IGHJ5 coding segment), both in non-malignant B cell samples. Each had sequence additions at the breakpoints albeit different from ours. Of follicular and diffuse large B cell lymphoma entries in the database, 163 had a breakpoint within 1Kb of our der(14) IGHJ5 breakpoint, with 34 matching within 5 bp. We did not find exact, or as nearly exact, breakpoint matches in the database for other t(14;18) junctional sequences. For the der(18) IGHD5-12 breakpoint the nearest entry was 9 bp away for a non-malignant B cell sample and 1840 bp away for a B cell lymphoma. For our breakpoints centromeric of *BCL-2*, the nearest entry, from a B cell lymphoma, was 683 and 695 bp away from our der(14) and der(18) breakpoints, respectively. In a pattern similar to dbCRID, 90% of *BCL-2* breakpoints in the database were in the 3′ UTR, the vast majority clustering in a 150 bp window. Only 3% of *BCL-2* breakpoints were further downstream of the gene than ours. Nonetheless our der(14) conformed in structure to the predominant der(14) model from the database, whereby the IGHJ locus adjoins *BCL-2*, coding sequence intact.

Because the IGH-*BCL-2* translocation had not to our knowledge been reported previously in this cell line, we performed standard metaphase karyotyping analysis. Seventeen metaphases were evaluated in this near tetraploid cell line. The cells showed the following composite, complex karyotype: 77 ~ 80 < 4n>, XXYY, -1, -2, -3, -3, -4, add(4)(p15.2), -5, add(5)(p13), del(5)(q22q33), -6,+7, der(8;?19)(p10;?q10), -9, -9, -10, add(10)(q22), -12, -13, -13, add(13)(q34), -14, t(14;18)(q32;q21), -15, -16, der(18)t(14;18)(q32;q21), +20, add(22)(q11.2), +mar1, +mar2[cp17]. The abnormalities observed in the karyotype aside from t(14;18) were not seen in the sequencing data as we had no baits covering these loci.

## Discussion

**Figure 6:**
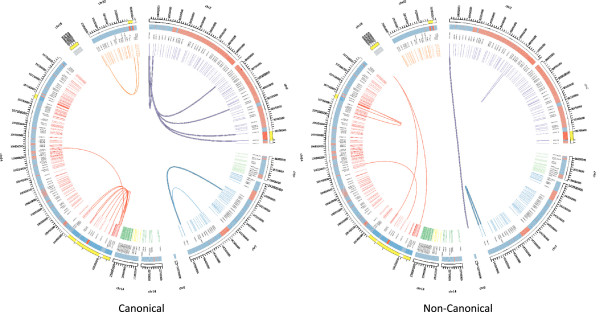
**Observed V(D)J recombination events.** Chromosome positions are shown in the outermost curved segments, one segment per contiguous genomic interval. Alignability is represented by red and blue tiles directly within, blue for regions that are > = 80% alignable and red for regions that are < 80% alignable, given 100 bp reads. Colored slashes composing the innermost ring represent VDJ segments. Different colors represent different loci, with the name of the locus and the segment appearing between the slashes and the curved segments. Line segments within the circle represent observed recombinations of VDJ elements or VDJ elements with non-VDJ sequence as with the chromosome translocations. The highlighted sections are areas over which the scale has been changed to better distinguish the recombination events. Taller segments with black borderlines represent inversions.

We have designed and implemented a method for interrogating V(D)J segment rearrangement using a targeted, sequence-specific capture method combined with Illumina sequencing. This method can detect non-canonical as well as canonical V(D)J recombination, even within segmental duplications. We have also provided evidence for single base resolution at the breakpoints, as indicated by Sanger sequencing validation of split-reads mappings (Additional files 2 and 3). In aggregate, we report 8 non-canonical structural rearrangements across 6 samples, two of which have been previously reported, as well as 18 canonical rearrangements (Figure 6). Excluding events within IGH, most of the canonical arrangements were inversions, with kappa light chain V to J rearrangement predominating (Figure 2, Additional file 1: Figure S1). This may reflect bias in our enrichment strategy, which placed baits for sequence recovery on signal joint sides of RSSs. Symmetric bait coverage surrounding RSSs would be expected to mitigate this bias.

A relatively large portion of events was from the LCL sample. This is consistent with the oligoclonal or heterogeneous derivation of this cell line, which provides more opportunity to detect events than clonal, neoplastic cells. The finding suggests that sequence capture methods may be applied to mixed lymphoid populations, including oligoclonal proliferations in the context of disease or polyclonal populations at various stages of lymphoid development. Understanding detection limits in populations of cells and applying the method to single cells are areas of future investigation.

For all junctional reads from coding segments, we observed N-region addition of nucleotides at the breakpoints, which we attribute to terminal deoxynucleotidyl transferase (TdT) enzyme activity (Figures 3 and 5 B, Additional file 1: Figures S2, S4 and S5). We did not see this same addition for junctions overlapping only RSS elements, with RSS-RSS junctions mapping exactly to the reference genome with no intervening, untemplated bases (Figures 2 and 4, Additional file 1: Figures S1 and S6). Most nucleotide additions adjacent to a coding segment were between 2 and 5 bases, although we observed 3 longer additions. For one of these exceptions, a recombination by interstitial deletion, we observed 23 N-region nucleotides that did not align to either side of the juxtaposed sequences (Additional file 1: Figure S5). Although this is an unusual length of nucleotides for TdT addition, it is unclear if dysregulated TdT activity is otherwise related to the proliferation.

We expect that inspection of V(D)J recombination using high throughput sequencing will provide a more complete picture of rearrangement at antigen receptor loci. Perhaps more importantly, next generation capture sequencing approaches such as ours may prove powerful in the study of how lymphoid populations change in response to antigenic selection or during clonal evolution of malignancies [18].

## Conclusions

Our experience underscores the value of targeted methods, both in sequencing and in analytical approaches. First, we have optimized cost and output with a sequence-based targeted pull down method, enabling deep sequencing of selected regions while minimizing data generation and processing time. Excellent coverage of desired sequences with effective exclusion of the remainder of the genome demonstrates the exquisite specificity of RSS sequences for genomic DNA capture. Secondly, in analytical aspects, we have balanced throughput with accuracy and shown the utility of contextual inspections of ambiguous aligning sequences.

Other methods of sequencing products of V(D)J recombination, even those leveraging considerable sequencing throughput, frequently focus on expressed products present in RNA/cDNA fractions or products that are amplifiable from genomic DNA sequences using primers anticipating canonical rearrangements of coding segments. Our method has the advantage of independence from these. As such, it should prove especially useful for finding off target and non-canonical rearrangements, such as the V(D)J recombinase-mediated chromosomal translocations and non-canonical V-V deletion events we described here. The latter example, which occurred with unexpected frequency in our small sample set, reflects a category of events that have not been well described outside of rearranged V-replacement. Finally, this advantage of our approach may facilitate the detection of V(D)J recombinase-mediated translocations in preneoplastic leukemia samples [19] or the identification of transposition events [20,21] in lymphocyte development and in lymphoid pathologies.

## Methods

### SureSelect library design

Recombination signal sequences (RSSs) recognized by RAG recombinase were downloaded from the international ImMunoGeneTics (IMGT) database [22] and used to recover corresponding genomic coordinates by alignment to the human reference assembly (hg19/GRCh37, February 2009) with Blat [23]. For each matching sequence, a 200 bp target window immediately flanking V(D)J coding segments was defined, extending across the entire RSS and into non-coding sequence 3′ of the RSS; these were merged to 440 genomic intervals for five-fold Agilent SureSelect bait tiling (Agilent Technologies, Santa Clara CA). A total of 2461 120-mer baits were designed to pull-down these sequences using eArray (Additional file 4).

### Sample collection

The Johns Hopkins Medicine Institutional Review Board granted approval for the study with reference number NA_00050660. Kathleen H. Burns, a clinical hematopathologist and co-author of this work, acquired cells from the clinical flow cytometry lab at Johns Hopkins Hospital. Samples were collected for diagnostic purposes with research material appropriated from the excess of what was needed for diagnostics. No additional proce-dures were performed to collect these samples, and so there was no associated medical risk to patients. Aliquots used for this study would have otherwise been discarded from the clinical flow cytometry lab. Samples were de-identified so that no patient information would follow samples to the lab. The Johns Hopkins Institutional Review Board approved their use without patient consent.

### Sample description

We identified rearrangements in four primary lymphoid malignancies; four cancer cell lines (American Type Culture Collection, Manassas, VA); and one EBV-transformed lymphoblastoid cell line (HuRef, Coriell Institute, Camden, NJ). No rearrangements were identified in two primary lymphoid malignancies studied, a chronic lymphocytic leukemia and a precursor T-cell acute lymphoblastic leukemia. The four primary neoplasias included a precursor B-cell acute lymphoblastic leukemia; a post transplant lymphoproliferative disorder consistent with a high grade, Burkitt-like lymphoma; a mantle cell lymphoma; and a chronic lymphocytic leukemia of the T-cell lineage. The four cell lines included the Ramos Burkitt lymphoma line; the DB diffuse large B-cell lymphoma of follicle center cell phenotype line; the ARH-77 plasma cell leukemia line; and Loucy acute precursor T-cell lymphoblastic lymphoma line.

### Targeted DNA library preparation and sequencing

High molecular weight gDNA was obtained from viable cells or fresh frozen primary cells by phenol-chloroform extraction and ethanol precipitation. For each sample, we sonicated gDNA to 500 bp (median size). Sample DNA was end-repaired using NEBNext End Repair Module (New England Biolabs, Ipswich MA), purified, dA-tailed, and ligated to index-specific, paired-end adapters from Illumina’s Multiplexing Oligonucleotide Kit (Illumina Inc, San Diego CA). Agilent’s SureSelect Target Enrichment System Kit for Illumina Paired-End Multiplexed Sequencing was used to complete library preparation and pull-down. Adapter-ligated DNA samples were PCR-amplified, purified, and hybridized to custom-designed RNA baits to capture our targeted DNA. Captured DNA was pulled down using Dynal MyOne Streptavidin T1 magnetic beads (Life Technologies, Carlsbad CA). Pulled-down DNA samples were index tagged using Illumina’s Multiplexing Oligonucleotide Kit. Products were purified with AMPure XP magnetic beads to produce targeted libraries. HudsonAlpha Institute for Biotechnology (Huntsville, AL) performed quality control, pooling, and sequencing of our indexed samples. The multiplexed library was sequenced in one lane of an Illumina HiSeq generating 7,339,278 100 bp paired-end reads of median insert size 254 and median coverage 173X across the 440 bait regions.

### Rearrangement identification

We aligned reads to hg19, used HYDRA to identify candidate recombinations [24], and visualized sites using the Integrative Genomics Viewer [25]. Breakpoints were determined by splitting reads with at least one member of a pair mapping to one of the two loci for an event, and remapping. We visualized split-reads using Jalview [26].

### Validation

We designed primers to amplify across rearrangement breakpoints, calling an event validated if we observed the expected band and no similarly sized band within control DNA. We validated all reported non-canonical and some canonical events (Additional files 2, 3, 5).

### Histopathology and flow cytometry

Morphologic features of primary tumors were studied by hematoxylin and eosin staining of tissue sections. Phenotyping by immunohistochemistry or flow cytometry was performed in the clinical laboratories at the Johns Hopkins Hospital as part of the routine diagnostic work-up. Lesions were classified according to the 2008 World Health Organization diagnostic criteria.

## Abbreviations

RSS: Recombination signal sequence; Ig: Immunoglobulin; TCR: T-cell receptor; TRB: T-cell receptor β chain; IGK: Immunoglobulin kappa light chain; IGL: Immunoglobulin lambda light chain; IGH: Immunoglobulin heavy chain; LCL: Lymphoblastoid cell line; EBV: Epstein-Barr virus; TdT: terminal deoxynucleotidyl transferase.

## Competing interests

The authors declare no competing financial interests.

## Authors’ contributions

KB, TF, and NG-C designed the study. NG-C and JS performed the experiment. EHS provided analytical tools, analyzed the data, and wrote the manuscript. EHS and JS performed validation. EHS, KB, JS, and SD interpreted results and edited the manuscript. All authors read and approved the final manuscript.

## Supplementary Material

Additional file 1: Figure S1 A canonical V-J rearrangement of the kappa immunoglobulin light chain locus in the Burkitt-like lymphoma. The rearrangement occurred by inversion of a 740 KB segment of 2p11.2. **Figure S2.** A canonical V(D)J rearrangement of the TRB locus in the Loucy cell line. The rearrangement occurred in 7q34 with deletion between two facing RSSs from TRBJ2-1 and an apparently un-annotated TRBD element (but with matches in the IMGT sequence database). The deletion was followed by an inversion between TRBV5-6 and the recombined D-J segments. **Figure S3.** An interstitial deletion within the IGK locus in LCL. The deletion encompasses 11 kb between two adjacent tandemly oriented V segments. **Figure S4.** An interstitial deletion within the IGH locus variable domain of the LCL. The deletion involves a 5 kb segment flanked by two RSS sequences with 23 bp spacers (filled arrows). The deletion was likely mediated through V replacement, although this allele has not undergone prior rearrangement at this locus. **Figure S5.** An interstitial deletion within the IGH locus variable domain of the ARH-77 cell line. The deletion involves a 5 kb segment flanked by two RSS sequences with 23bp spacers (filled arrows). The deletion was likely mediated through V replacement, although this allele has not undergone prior rearrangement at this locus. **Figure S6.** An inappropriate V-J recombination of at the TRB locus in chronic T-cell leukemia. This locus should include a D element. We did not observe the V-J segment joining directly but we infer from the presence of a J element RSS, which should have been lost given a canonical V(D)J rearrangement, that his event is non-canonical. The rearrangement occurred by inversion of a 16 KB segment of 7q34. Click here for file

Additional file 2 Split read sequences (matching those in main and supplemental figures) with corresponding Sanger sequences. Click here for file

Additional file 3 Validation strategy using PCR and Sanger sequencing. Click here for file

Additional file 4 RSS genomic coordinates used to design probes. Click here for file

Additional file 5 Detected Rearrangements, each row representing one side of a junction. Click here for file

## References

[B1] Alamyar E Duroux P Lefranc MP Giudicelli V IMGT((R)) tools for the nucleotide analysis of immunoglobulin (IG) and T cell receptor (TR) V-(D)-J repertoires, polymorphisms, and IG mutations: IMGT/V-QUEST and IMGT/HighV-QUEST for NGS Methods Mol Biol 2012 882 569 604 10.1007/978-1-61779-842-9_32 22665256

[B2] Lefranc MP Giudicelli V Busin C Bodmer J Muller W Bontrop R Lemaitre M Malik A Chaume D IMGT, the International ImMunoGeneTics database Nucleic Acids Res 1998 26 1 297 303 10.1093/nar/26.1.297 9399859 PMC147225

[B3] Fischer N Sequencing antibody repertoires: the next generation mAbs 2011 3 1 17 20 10.4161/mabs.3.1.14169 21099370 PMC3038007

[B4] Watson CT Breden F The immunoglobulin heavy chain locus: genetic variation, missing data, and implications for human disease Genes Immun 2012 13 5 363 373 10.1038/gene.2012.12 22551722

[B5] Nilsson K Klein G Phenotypic and cytogenetic characteristics of human B-lymphoid cell lines and their relevance for the etiology of Burkitt’s lymphoma Adv Cancer Res 1982 37 319 380 6305160 10.1016/s0065-230x(08)60886-6

[B6] Wasserman R Ito Y Galili N Yamada M Reichard BA Shane S Lange B Rovera G The pattern of joining (JH) gene usage in the human IgH chain is established predominantly at the B precursor cell stage J Immunol 1992 149 2 511 516 1624797

[B7] Huber C Klobeck HG Zachau HG Ongoing V kappa-J kappa recombination after formation of a productive V kappa-J kappa coding joint Eur J Immunol 1992 22 6 1561 1565 10.1002/eji.1830220632 1601042

[B8] Malissen M McCoy C Blanc D Trucy J Devaux C Schmitt-Verhulst AM Fitch F Hood L Malissen B Direct evidence for chromosomal inversion during T-cell receptor beta-gene rearrangements Nature 1986 319 6048 28 33 10.1038/319028a0 3484541

[B9] Fanning L Bertrand FE Steinberg C Wu GE Molecular mechanisms involved in receptor editing at the Ig heavy chain locus Int Immunol 1998 10 2 241 246 10.1093/intimm/10.2.241 9533453

[B10] Szczepanski T Pongers-Willemse MJ Langerak AW Harts WA Wijkhuijs AJ Van Wering ER Van Dongen JJ Ig heavy chain gene rearrangements in T-cell acute lymphoblastic leukemia exhibit predominant DH6-19 and DH7-27 gene usage, can result in complete V-D-J rearrangements, and are rare in T-cell receptor alpha beta lineage Blood 1999 93 12 4079 4085 10361104

[B11] Born W Yague J Palmer E Kappler J Marrack P Rearrangement of T-cell receptor beta-chain genes during T-cell development Proc Natl Acad Sci USA 1985 82 9 2925 2929 10.1073/pnas.82.9.2925 3873070 PMC397679

[B12] Kroenlein H Schwartz S Reinhardt R Rieder H Molkentin M Gokbuget N Hoelzer D Thiel E Burmeister T Molecular analysis of the t(2;8)/MYC-IGK translocation in high-grade lymphoma/leukemia by long-distance inverse PCR Genes Chromosome Canc 2012 51 3 290 299 10.1002/gcc.21915 22120970

[B13] Lipford E Wright JJ Urba W Whang-Peng J Kirsch IR Raffeld M Cossman J Longo DL Bakhshi A Korsmeyer SJ Refinement of lymphoma cytogenetics by the chromosome 18q21 major breakpoint region Blood 1987 70 6 1816 1823 2823937

[B14] Bakhshi A Wright JJ Graninger W Seto M Owens J Cossman J Jensen JP Goldman P Korsmeyer SJ Mechanism of the t(14;18) chromosomal translocation: structural analysis of both derivative 14 and 18 reciprocal partners Proc Natl Acad Sci U S A 1987 84 8 2396 2400 10.1073/pnas.84.8.2396 3104914 PMC304658

[B15] Kong F Zhu J Wu J Peng J Wang Y Wang Q Fu S Yuan LL Li T dbCRID: a database of chromosomal rearrangements in human diseases Nucleic Acids Res 2011 39 Database issue D895 D900 21051346 10.1093/nar/gkq1038 PMC3013658

[B16] Matolcsy A Casali P Warnke RA Knowles DM Morphologic transformation of follicular lymphoma is associated with somatic mutation of the translocated Bcl-2 gene Blood 1996 88 10 3937 3944 8916960

[B17] Tsai AG Lu H Raghavan SC Muschen M Hsieh CL Lieber MR Human chromosomal translocations at CpG sites and a theoretical basis for their lineage and stage specificity Cell 2008 135 6 1130 1142 10.1016/j.cell.2008.10.035 19070581 PMC2642632

[B18] Robison K Application of second-generation sequencing to cancer genomics Brief Bioinform 2010 11 5 524 534 10.1093/bib/bbq013 20427421

[B19] Wiemels JL Cazzaniga G Daniotti M Eden OB Addison GM Masera G Saha V Biondi A Greaves MF Prenatal origin of acute lymphoblastic leukaemia in children Lancet 1999 354 9189 1499 1503 10.1016/S0140-6736(99)09403-9 10551495

[B20] Agrawal A Eastman QM Schatz DG Transposition mediated by RAG1 and RAG2 and its implications for the evolution of the immune system Nature 1998 394 6695 744 751 10.1038/29457 9723614

[B21] Hiom K Melek M Gellert M DNA transposition by the RAG1 and RAG2 proteins: a possible source of oncogenic translocations Cell 1998 94 4 463 470 10.1016/S0092-8674(00)81587-1 9727489

[B22] Giudicelli V Duroux P Ginestoux C Folch G Jabado-Michaloud J Chaume D Lefranc MP IMGT/LIGM-DB, the IMGT comprehensive database of immunoglobulin and T cell receptor nucleotide sequences Nucleic Acids Res 2006 34 Database issue D781 D784 16381979 10.1093/nar/gkj088 PMC1347451

[B23] Kent WJ BLAT–the BLAST-like alignment tool Genome Res 2002 12 4 656 664 11932250 10.1101/gr.229202 PMC187518

[B24] Quinlan AR Clark RA Sokolova S Leibowitz ML Zhang Y Hurles ME Mell JC Hall IM Genome-wide mapping and assembly of structural variant breakpoints in the mouse genome Genome Res 2010 20 5 623 635 10.1101/gr.102970.109 20308636 PMC2860164

[B25] Robinson JT Thorvaldsdottir H Winckler W Guttman M Lander ES Getz G Mesirov JP Integrative genomics viewer Nat Biotechnol 2011 29 1 24 26 10.1038/nbt.1754 21221095 PMC3346182

[B26] Waterhouse AM Procter JB Martin DM Clamp M Barton GJ Jalview Version 2–a multiple sequence alignment editor and analysis workbench Bioinformatics 2009 25 9 1189 1191 10.1093/bioinformatics/btp033 19151095 PMC2672624

