# Supplementary material for: Fine mapping of V(D)J recombinase mediated rearrangements in human lymphoid malignancies
Source: BMC Genomics. 2013 Aug 19;14:565. doi: 10.1186/1471-2164-14-565 (PMC3846541; doi:10.1186/1471-2164-14-565)
Supplement: Additional file 1: Figure S1 — A canonical V-J rearrangement of the kappa immunoglobulin light chain locus in the Burkitt-like lymphoma. The rearrangement occurred by inversion of a 740 KB segment of 2p11.2. Figure S2. A canonical V(D)J rearrangement of the TRB locus in the Loucy cell line. The rearrangement occurred in 7q34 with deletion between two facing RSSs from TRBJ2-1 and an apparently un-annotated TRBD element (but with matches in the IMGT sequence database). The deletion was followed by an inversion between TRBV5-6 and the recombined D-J segments. Figure S3. An interstitial deletion within the IGK locus in LCL. The deletion encompasses 11 kb between two adjacent tandemly oriented V segments. Figure S4. An interstitial deletion within the IGH locus variable domain of the LCL. The deletion involves a 5 kb segment flanked by two RSS sequences with 23 bp spacers (filled arrows). The deletion was likely mediated through V replacement, although this allele has not undergone prior rearrangement at this locus. Figure S5. An interstitial deletion within the IGH locus variable domain of the ARH-77 cell line. The deletion involves a 5 kb segment flanked by two RSS sequences with 23bp spacers (filled arrows). The deletion was likely mediated through V replacement, although this allele has not undergone prior rearrangement at this locus. Figure S6. An inappropriate V-J recombination of at the TRB locus in chronic T-cell leukemia. This locus should include a D element. We did not observe the V-J segment joining directly but we infer from the presence of a J element RSS, which should have been lost given a canonical V(D)J rearrangement, that his event is non-canonical. The rearrangement occurred by inversion of a 16 KB segment of 7q34. [file 1471-2164-14-565-S1.pdf]

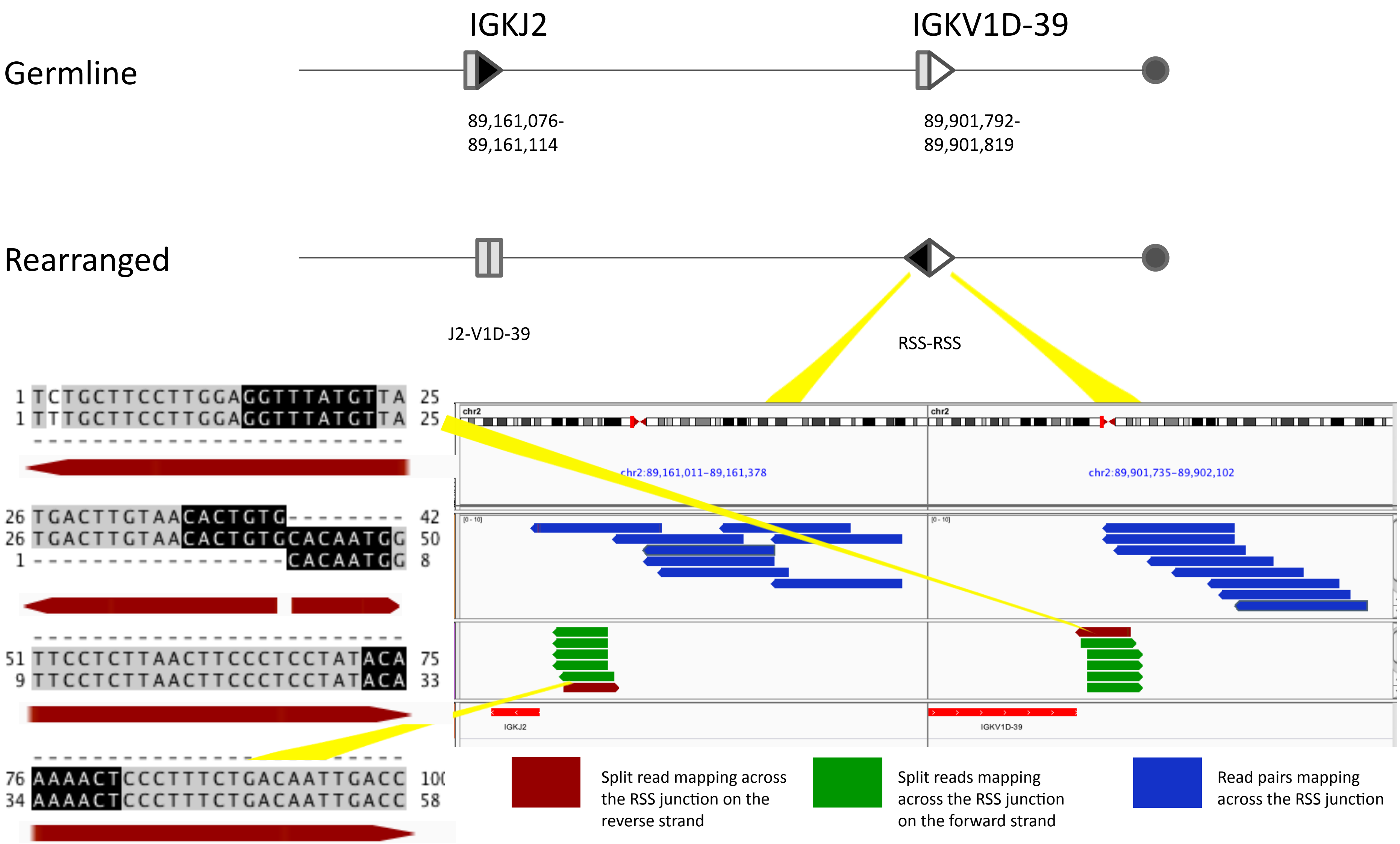

Row 1: Reverse strand IGKV1D-39 RSS locus (reverse complement heptamer and nonamer in black)
Row 2: Split read
Row 3: Forward strand IGKJ2 RSS locus (heptamer and nonamer in black)

Supplemental Figure 1

Figure S1
A canonical V-J rearrangement of the kappa immunoglobulin light chain locus in the Burkitt-like lymphoma. The rearrangement occurred by inversion of a 740 KB segment of 2p11.2. A schematic of the initial allele and the resulting product of the recombination are shown at the top. V and J segments are shown as gray boxes; recombination signal sequences (RSSs) are shown with arrows and marked with their corresponding genome coordinates. Arrow orientation reflects the direction of the RSS (heptamer-to-nonamer), filled arrows illustrate RSS with 23bp (two turn) spacers, and open arrows illustrate 12bp (one turn) spacers. The centromere side is marked with a circle, not distanced to scale. The RSS-RSS junction was detected by the sequencing strategy (right lower panel); blue reads denote pairs mapping across the junction. V and J are shown in red below the reads. The rearrangement was confirmed by Sanger sequencing, with the recovered sequence matching the junction spanning sequence found with a split-read (left lower panel, middle sequence). The split-read sequence is shown aligned to the two sequences present in the reference genome (hg19). Nucleotides aligning to the reference regions are shown in gray, a mismatch between the sample and reference is shown in white, and heptamers and nonamers are shown in black. The IGKV1D-39 RSS is shown reverse complemented while the IGKJ2 RSS is shown in proper 5’ to 3’ orientation, reflecting the nature of a split-read over an inversion.

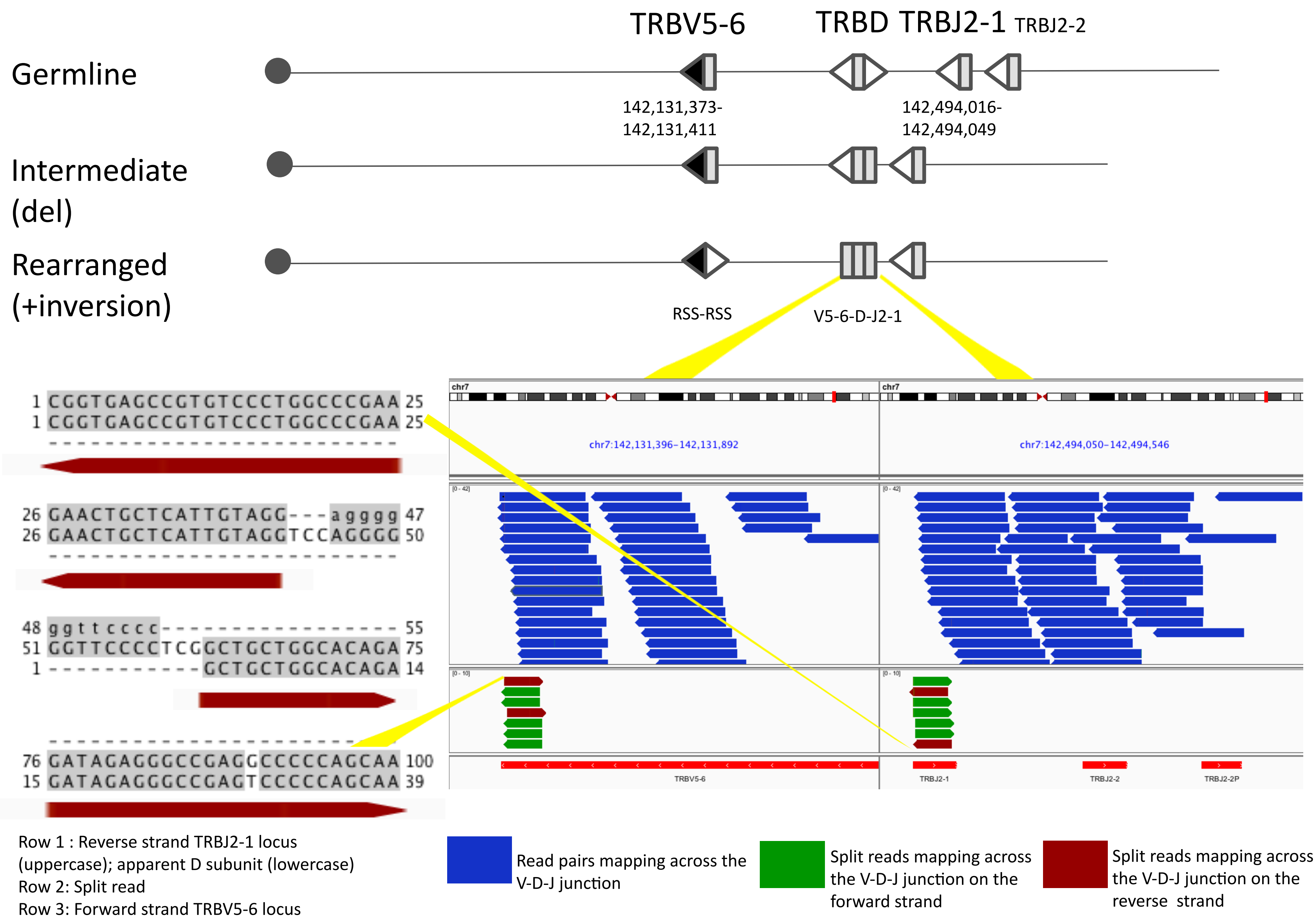

Supplemental Figure 2

Figure S2

A canonical V(D)J rearrangement of the TRB locus in the Loucy cell line. The rearrangement occurred in 7q34 with deletion between two facing RSSs from TRBJ2-1 and an apparently un-annotated TRBD element (but with matches in the IMGT sequence database). The deletion was followed by an inversion between TRBV5-6 and the recombined D-J segments. A schematic of the initial allele and the resulting product of the recombination are shown at the top. V,D, and J segments are shown as gray boxes; recombination signal sequences (RSSs) are shown with arrows and marked with their corresponding genome coordinates. Arrow orientation reflects the direction of the RSS (heptamer-to-nonamer), filled arrows illustrate RSS with 23bp (two turn) spacers, and open arrows illustrate 12bp (one turn) spacers. The centromere side is marked with a circle, not distanced to scale. The V-D-J junction was detected by the sequencing strategy (right lower panel); blue reads denote pairs mapping across the junction. V and J segments are shown in red below the reads. The rearrangement was confirmed by Sanger sequencing, with the recovered sequence matching the junction spanning sequence found with a split-read (left lower panel, middle sequence). The split-read sequence is shown aligned to the two sequences present in the reference genome (hg19). The un-annotated D element is shown in lowercase on row 1. Nucleotides aligning to the reference regions and D element are shown in gray, N-region nucleotides and a mismatch between the sample and the reference are white.

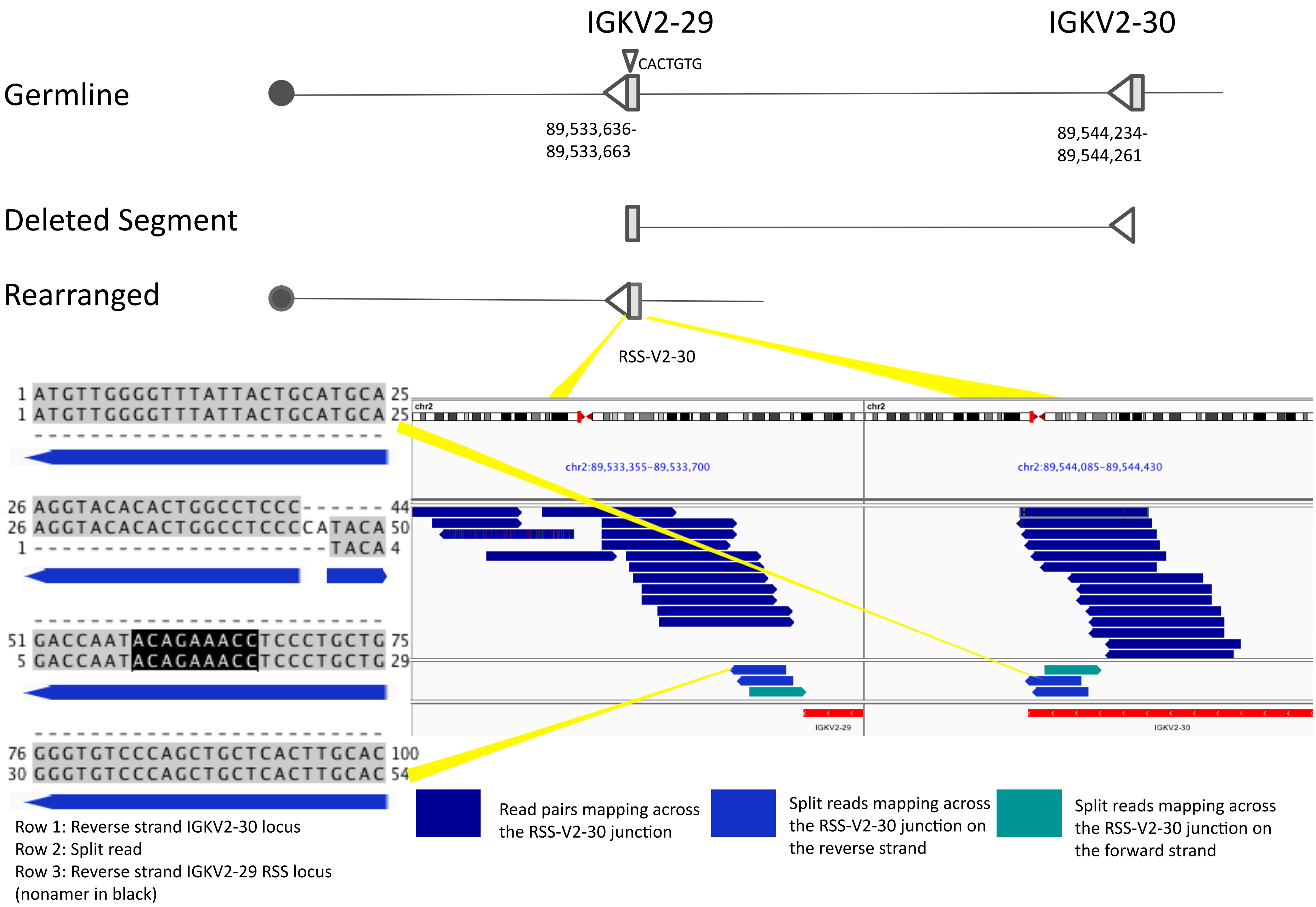

Supplemental Figure 3

Figure S3

An interstitial deletion within the IGK locus in LCL. The deletion encompasses 11kb between two adjacent tandemly oriented V segments. Open arrows represent 12bp spacer variable domain RSSs. The deletion was likely mediated through V replacement, although this allele has not undergone prior rearrangement at this locus. The deletion is initiated through recognition of an oppositely oriented cryptic heptamer (shown as a small downward facing, open triangle) at the signal flank of IGKV2-29 by the RSS of IGKV2-30. In this case the cryptic heptamer is the reverse complement of the canonical 12bp spacer RSS heptamer. The deletion was detected by sequencing (lower right panel); blue reads denote sequences with read pairs mapping across the junction. The rearrangement was confirmed by Sanger sequencing, with the recovered sequence matching the junction spanning sequence found with a split-read (left lower panel, middle sequence). The split-read sequence is shown aligned to the two sequences present in the reference genome (hg19). Nucleotides aligning to the reference regions are shown in gray, N-region nucleotides are white, and the retained nonamer of IGKV2-29 is shown in black. Other interstitial deletions in this locus are known to be variant germline alleles, though this rearrangement is not reported as such and may be somatic.

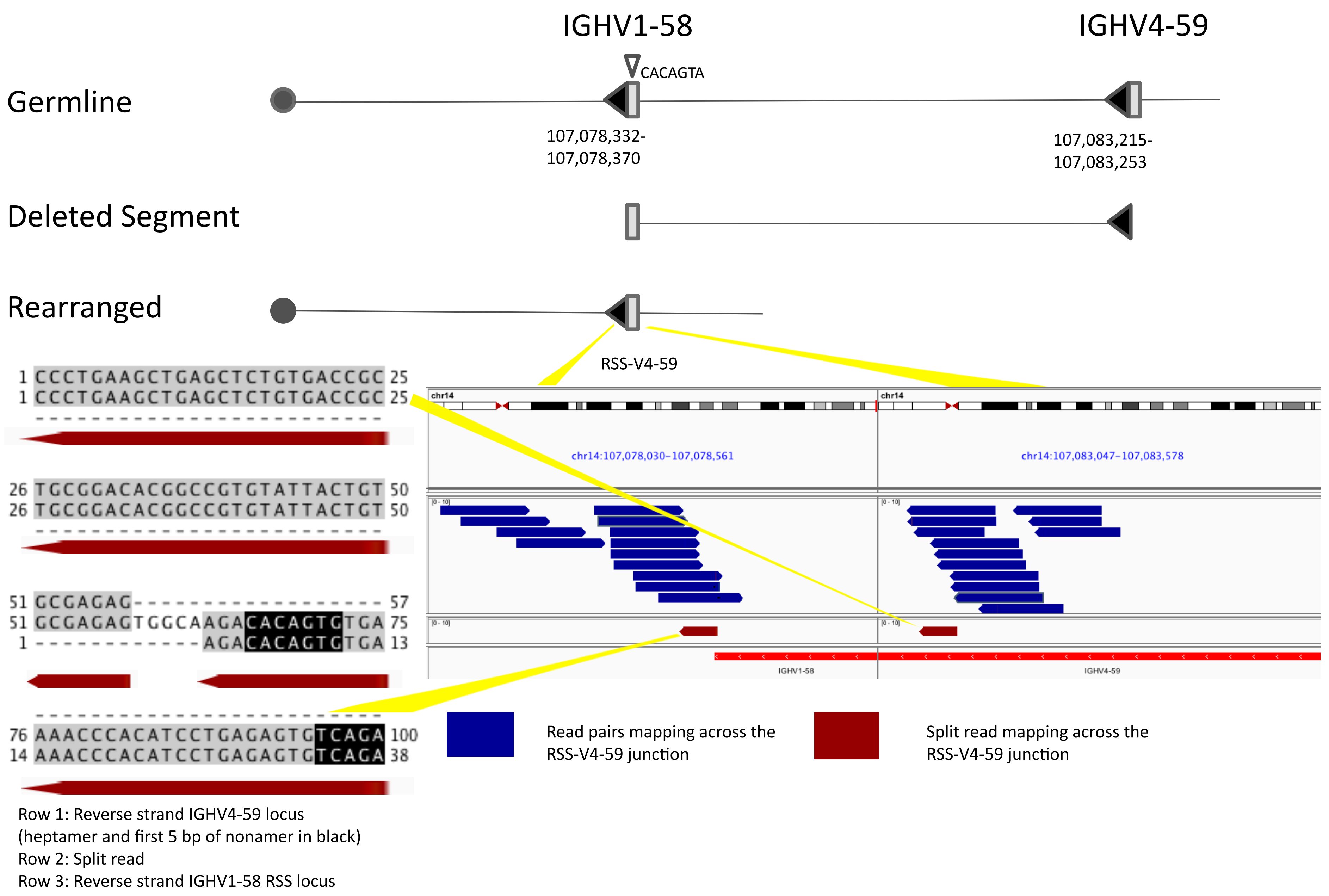

Supplemental Figure 4

Figure S4

An interstitial deletion within the IGH locus variable domain of the LCL. The deletion involves a 5kb segment flanked by two RSS sequences with 23bp spacers (filled arrows). The deletion was likely mediated through V replacement , although this allele has not undergone prior rearrangement at this locus. The deletion is initiated through recognition of an oppositely oriented cryptic heptamer (shown as a small downward facing, open triangle) within IGHV1-58 by the RSS of IGHV4-59. The deletion was detected by sequencing of the RSS-V4-59 junction (lower right panel); blue reads denote sequences with read pairs mapping across the junction. The rearrangement was confirmed by Sanger sequencing, with the recovered sequence matching the junction spanning sequence found with a split-read (left lower panel, middle sequence). The split-read sequence is shown aligned to the two sequences present in the reference genome (hg19). Nucleotides aligning to the reference regions are shown in gray, N-region nucleotides are white, and the retained heptamer and nonamer of IGHV1-58 are shown in black. The split-read was long enough only to capture the first 5 bases of the nonamer.

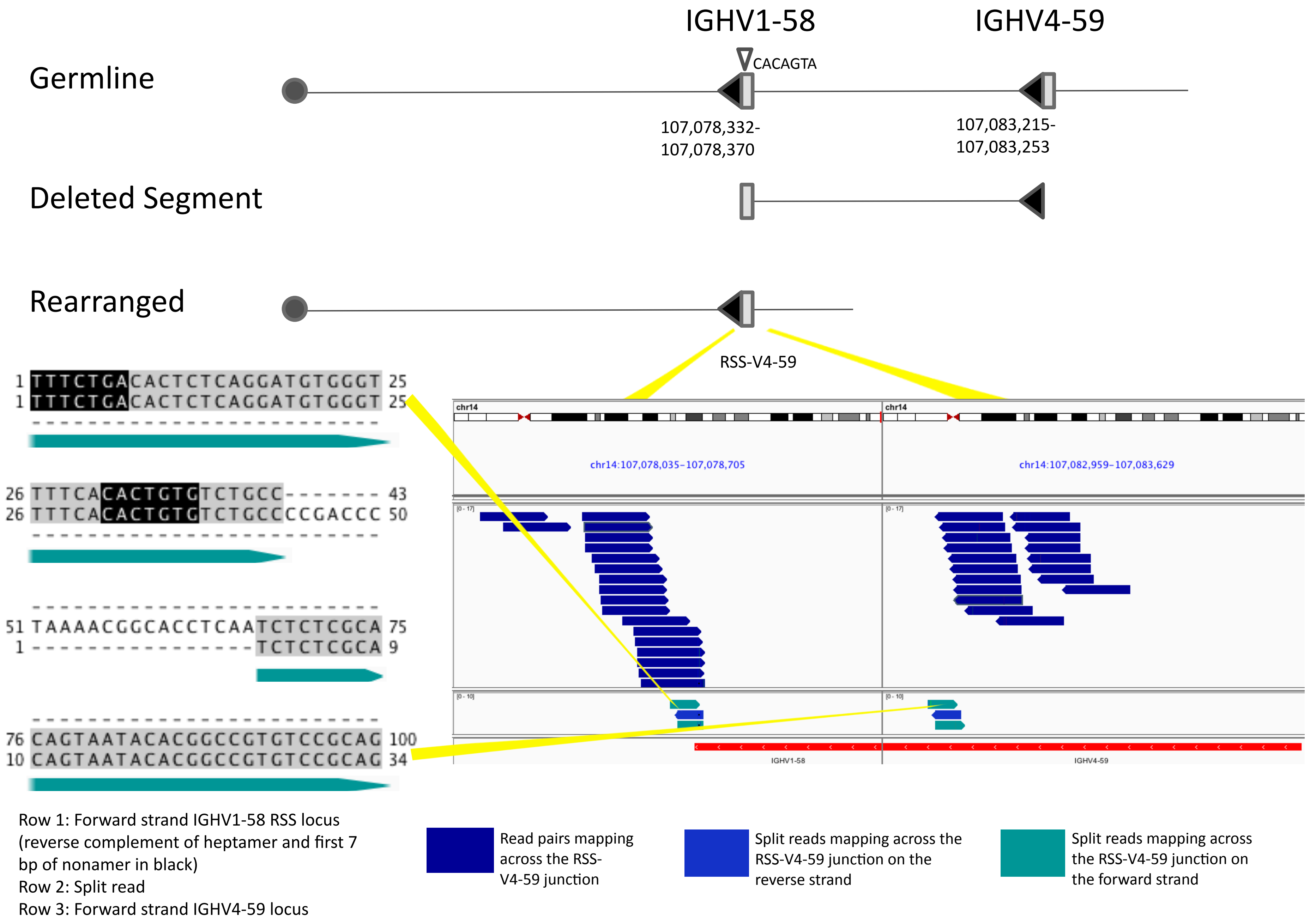

Supplemental Figure 5

Figure S5
An interstitial deletion within the IGH locus variable domain of the ARH-77 cell line. The deletion involves a 5kb segment flanked by two RSS sequences with 23bp spacers (filled arrows). The deletion was likely mediated through V replacement , although this allele has not undergone prior rearrangement at this locus. The deletion is initiated through recognition of an oppositely oriented cryptic heptamer (shown as a small downward facing, open triangle) within IGHV1-58 by the RSS of IGHV4-59 The deletion was detected by sequencing of the RSS-V segment junction (lower right panel); blue reads denote sequences with read pairs mapping across the junction. The rearrangement was confirmed by Sanger sequencing, with the recovered sequence matching the junction spanning sequence found with a split-read (left lower panel, middle sequence). The split-read sequence is shown aligned to the two sequences present in the reference genome (hg19). Nucleotides aligning to the reference regions are shown in gray, N-region nucleotides are white, and the reverse complement of the retained heptamer and nonamer of IGHV1-58 are shown in black. The split-read was long enough only to capture the first 7 bases of the nonamer.

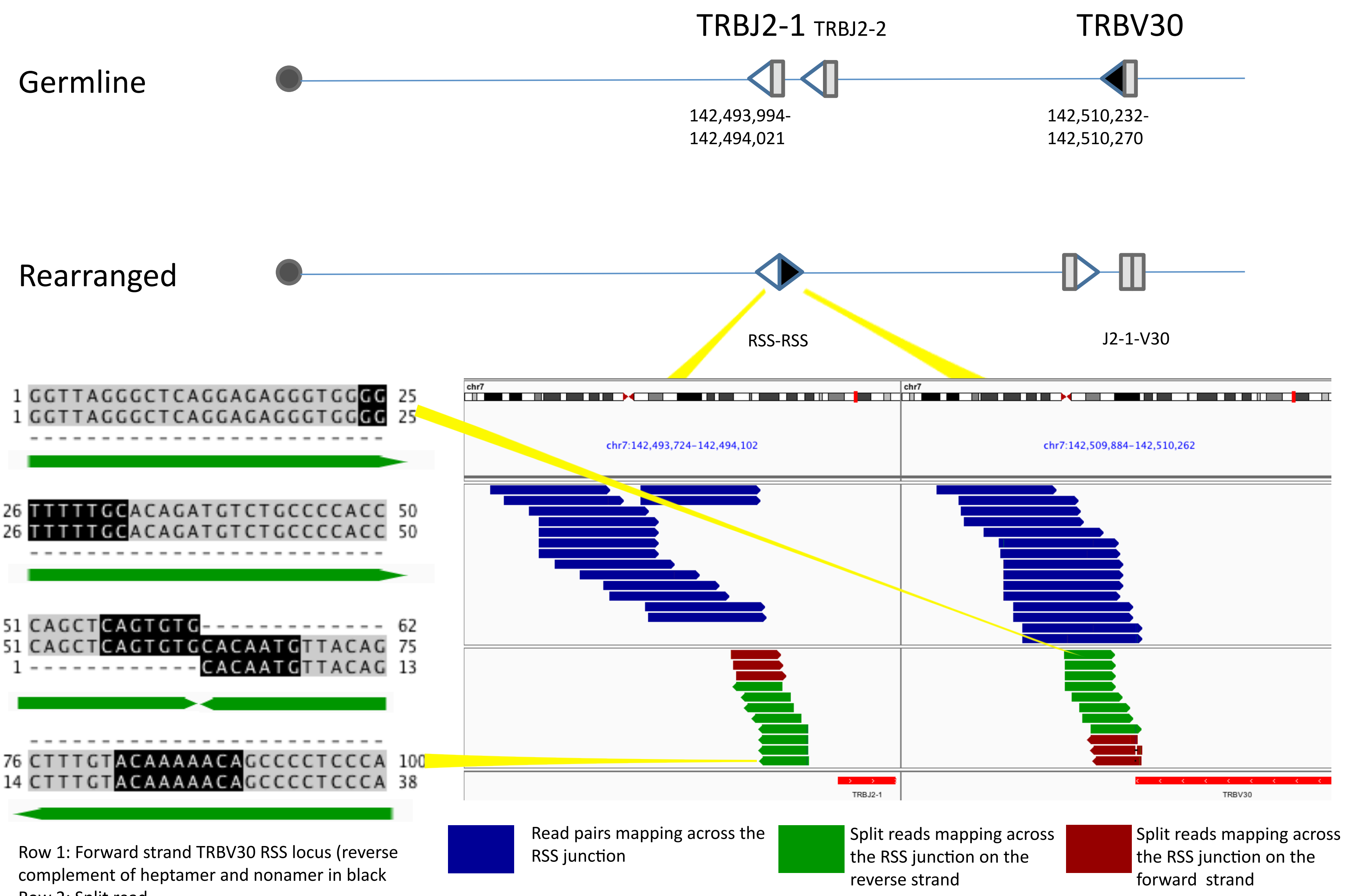

Row 1: Forward strand TRBV30 RSS locus (reverse complement of heptamer and nonamer in black
Row 2: Split read
Row 3: Reverse strand TRBJ2-1 RSS locus (heptamer and nonamer in black)

Supplemental Figure 6

Figure S6
An inappropriate V-J recombination of at the TRB locus in chronic T-cell leukemia. This locus should include a D element. We did not observe the V-J segment joining directly but we infer from the presence of a J element RSS, which should have been lost given a canonical V(D)J rearrangement, that his event is non-canonical. The rearrangement occurred by inversion of a 16 KB segment of 7q34. A schematic of the initial allele and the resulting product of the recombination are shown at the top. V and J segments are shown as gray boxes; recombination signal sequences (RSSs) are shown with arrows and marked with their corresponding genome coordinates. Arrow orientation reflects the direction of the RSS (heptamer-to-nonamer), filled arrows illustrate RSS with 23bp (two turn) spacers, and open arrows illustrate 12bp (one turn) spacers. The centromere side is marked with a circle, not distanced to scale. The RSS-RSS junction was detected by the sequencing strategy (right lower panel); blue reads denote pairs mapping across the junction. V and J segments are shown in red below the reads. The rearrangement was confirmed by Sanger sequencing, with the recovered sequence matching the junction spanning sequence found with a split-read (left lower panel, middle sequence). The split-read sequence is shown aligned to the two sequences present in the reference genome (hg19). Nucleotides aligning to the reference regions are shown in gray while heptamer and nonamer sequences are shown in black. reverse complement of the retained heptamer and nonamer of IGHV1-58 are shown in black. The TRBV30 RSS is shown reverse complemented while the TRBJ2-1 RSS is shown in proper 5’ to 3’ orientation, reflecting the nature of a split-read over an inversion.
