# Supplementary material for: Fine mapping of V(D)J recombinase mediated rearrangements in human lymphoid malignancies
Source: BMC Genomics. 2013 Aug 19;14:565. doi: 10.1186/1471-2164-14-565 (PMC3846541; doi:10.1186/1471-2164-14-565)
Supplement: Additional file 3 — Validation strategy using PCR and Sanger sequencing. [file 1471-2164-14-565-S3.docx]

Supplementary Methods: Validation Strategy

We designed primers to cross the breakpoints of non-canonical rearrangements predicted by >4 reads. This cutoff trimmed the list of candidates down from >12,000 to <200. For a negative control we used unrelated normal genomic DNA from peripheral blood mononuclear cells. We designed primers to cross breakpoints of a subset of canonical rearrangements as positive controls. We obtained PCR amplicons from our positive controls, comprising 6 junctions, and from 10 other candidate rearrangements, comprising 8 non-canonical recombination events, and both derivative chromosome junctions amplified for each of the 2 chromosomal translocations. For 12 out of 16 junctions, we also performed Sanger sequencing of the amplicons, including 9 junctions from non-canonical events (we sequenced junctions for both derivative chromosomes in the two chromosomal rearrangements) and 3 junctions from canonical inversions. The 4 junctions we validated but did not Sanger sequence were IGH D-J deletion rearrangements. All Sanger sequences verified correct, single base resolution of breakpoints using mapped split-reads.

Validation of a t(14;18) translocation in the DB cell line was performed in the clinical cytogenetics laboratory at the Johns Hopkins Hospital by standard karyotyping.
